# Biotransformation of Doxorubicin Promotes Resilience in Simplified Intestinal Microbial Communities

**DOI:** 10.1128/mSphere.00068-21

**Published:** 2021-05-26

**Authors:** Ryan A. Blaustein, Patrick C. Seed, Erica M. Hartmann

**Affiliations:** a Department of Civil and Environmental Engineering, Northwestern University, Evanston, Illinois, USA; b Feinberg School of Medicine, Northwestern University, Chicago, Illinois, USA; c Ann & Robert H. Lurie Children's Hospital of Chicago, Chicago, Illinois, USA; d The Stanley Manne Children’s Research Institute, Chicago, Illinois, USA; University of Wisconsin-Madison

**Keywords:** biotransformation, chemotherapeutic, doxorubicin, ecological resilience, gut microbiota

## Abstract

Chemotherapeutic drugs can cause harmful gastrointestinal side effects, which may be modulated by naturally occurring members of our microbiome. We constructed simplified gut-associated microbial communities to test the hypothesis that bacteria-mediated detoxification of doxorubicin (i.e., a widely used chemotherapeutic) confers protective effects on the human microbiota. Mock communities composed of up to five specific members predicted by genomic analysis to be sensitive to the drug or resistant via biotransformation and/or efflux were grown *in vitro* over three generational stages to characterize community assembly, response to perturbation (doxorubicin exposure), and resilience. Bacterial growth and drug concentrations were monitored with spectrophotometric assays, and strain relative abundances were evaluated with 16S rRNA gene sequencing. Bacteria with predicted resistance involving biotransformation significantly lowered concentrations of doxorubicin in culture media, permitting growth of drug-sensitive strains in monoculture. Such protective effects were not produced by strains with drug resistance conferred solely by efflux. In the mixed communities, resilience of drug-sensitive members depended on the presence and efficiency of transformers, as well as drug exposure concentration. Fitness of bacteria that were resistant to doxorubicin via efflux, though not transformation, also improved when the transformers were present. Our simplified community uncovered ecological relationships among a dynamic consortium and highlighted drug detoxification by a keystone species. This work may be extended to advance probiotic development that may provide gut-specific protection to patients undergoing cancer treatment.

**IMPORTANCE** While chemotherapy is an essential intervention for treating many forms of cancer, gastrointestinal side effects may precede infections and risks for additional health complications. We developed an *in vitro* model to characterize key changes in bacterial community dynamics under chemotherapeutic stress and the role of bacterial interactions in drug detoxification to promote microbiota resilience. Our findings have implications for developing bio-based strategies to promote gut health during cancer treatment.

## INTRODUCTION

Cancer treatment often involves a wide range of medications and additional intensive interventions. Chemotherapeutic agents lower mortality rates for many forms of cancer (1) but often fail to specifically target the cancerous tissue leading to toxicity to immune cells and the gut epithelium, among others. One major toxicity is mucositis of the enteric tract, where the epithelial barrier and its critical functions deteriorate, leading to pain, reduced absorption, ready translocation of enteric organisms, and associated inflammatory mediators (2, 3). Chemotherapeutics can further cause microbiome alterations that facilitate the emergence of antibiotic-resistant microorganisms and, especially for pediatric patients, increase risks for dysbiosis-related health complications later in life (e.g., obesity, asthma, and diabetes) (4, 5). Developing new strategies to mitigate the harmful consequences of chemotherapy on mucositis and dysbiosis would have far-reaching implications for improving health and well-being.

The intestinal microbiome plays important roles in modulating therapeutic outcomes through biotic and abiotic interactions (6, 7). For example, certain members of the gut microbiota have been found to activate an undesirable immune response linked to colorectal cancer chemoresistance (8). In contrast, disruption of commensal gut microbiota in antibiotic-treated mouse models was reported to limit the production of cytokines and reactive oxygen species that are critical for promoting tumor necrosis and limiting cytotoxicity during immunotherapy and chemotherapy, respectively (9). Moreover, natural bacterial enzymes can transform chemotherapeutics, antibiotics, and other medicinal agents to metabolites with altered toxicity (10, 11). For example, β-glucuronidase produced by microflora in the large intestine interacts with a metabolite of antitumor agent irinotecan hydrochloride (CPT-11) to cause dose-limiting gastrointestinal side effects (12). Alternatively, Raoultella planticola (i.e., a low-abundance gut commensal) was reported to deglycosylate the anticancer drug doxorubicin under anaerobic conditions (13). This biotransformation mechanism, which was also performed by strains of Escherichia coli and Klebsiella pneumoniae that had encoded the same biosynthetic pathway, yielded metabolites with reduced toxicity to the model eukaryote Caenorhabditis elegans (13). An additional aerobic NADH dehydrogenase-dependent doxorubicin transformation pathway that generates these same products has been described for *Streptomyces* sp. as well (14). Thus, while certain members of the human gut microbiota may have adverse impacts on treatment outcomes via complex interactions, preserving or promoting microbial diversity, among others, may be crucial for a positive host response.

Leveraging bacteria-mediated drug transformations may offer promise for managing unintended health risks of chemotherapeutic drugs. Beyond contributing to drug metabolism, biotransformations that limit chemical toxicity provide an ecological service to neighboring microbial cells (15). While it is well established that antibiotics impair gut microbiota diversity and enhance the dissemination of antibiotic resistance genes (16), chemotherapeutic drugs are known to perturb the microbiome as well (5). Potential effects of nonantibiotic compounds likely reflect their mechanism of action (e.g., DNA intercalation by anthracycline chemotherapeutics may have broad-spectrum toxicity to microbial cells in addition to targeted neoplastic tissue) and may select for drug-resistant strains (e.g., capable of multidrug export). Distinguishing specific effects of chemotherapy on the gut microbiome from prophylactic antibiotics is often confounded by usage together (5). Different mechanisms for antimicrobial resistance within a microbiota (e.g., drug efflux versus drug transformation) may have unique implications for responses among the broader community to perturbation. Whether resistance mechanisms enhance ecological resilience (i.e., the rate of recovery following a disturbance) (17) warrants investigation.

Modeling simplified mixed bacterial communities has enabled the characterization of key microbial and microbe-chemical interactions that may impact drug detoxification and/or host health (18–21). Cocultures can capture certain properties of more complex, naturally occurring microbiomes that are often challenging to investigate (19). Here, we constructed simplified human gut-associated microbial communities with a reductionist approach to test the hypothesis that bacteria-mediated transformation of doxorubicin promotes microbiota resilience. Our objectives were to (i) distinguish ways by which bacteria may resist the chemotherapy, and (ii) determine how different mechanisms of antimicrobial resistance may affect a broader microbial community. That is, while chemotherapeutic drugs may be toxic to the patient (e.g., epithelial damage) and the microbiome (i.e., antimicrobial effects), our *in vitro* model was designed to elucidate potential consequences to the latter that involve direct toxicity.

## RESULTS

### Doxorubicin resistance reflected bacterial genotype.

Clinical isolates representing five bacterial taxa with unique predicted responses to doxorubicin based on genomic analysis (e.g., drug resistance involving efflux and/or transformation) were selected for study as model members of the gut microbiota. The set included facultative and strict anaerobes with different human health implications (i.e., *Clostridium innocuum*, *Enterococcus faecium*, *Escherichia coli*, *Klebsiella pneumoniae*, and *Lactobacillus rhamnosus*) (Table 1). While some multidrug export proteins were encoded across all of the facultative strains (e.g., *yheI*), broader resistance profiles were encoded specifically by E. coli and K. pneumoniae (e.g., *mtdD* and *emrK*). The two latter strains also encoded a variety of molybdenum cofactor biosynthesis genes (22), which have been described to be essential for anaerobic deglycosylation of doxorubicin (13). E. faecium and the putative drug-sensitive strains C. innocuum and L. rhamnosus largely lacked such doxorubicin detoxification components.

**TABLE 1:** Bacterial taxa used in the *in vitro* model^*d*^

Bacterial strain or species	Phylum	Oxygen status	Gram status	Isolation (source)	Health implications	Genus frequency across gut microbiota (%)^*a*^	Multidrug export genes	Moco biosynthesis gene(s)^*b*^
C. innocuum ATCC 14501	*Firmicutes*	Anaerobe	Positive	Appendiceal abscess (Lurie Children's Hospital of Chicago)	Commensal; model for sporulation	89.5	*mdtK*, *mepA*, *norM*	*moaA*
L. rhamnosus	*Firmicutes*	Facultative	Positive	Vaginal swab (Washington University, St. Louis)	Commensal; probiotic	14.5	*emrB*, *mdtD mepA*, *yheH*, *yheI*	
E. faecium^*c*^	*Firmicutes*	Facultative	Positive	Bloodstream (Washington University, St. Louis)	Opportunistic pathogen	4.3	*emrB*, *yfmO*, *yheH*, *yheI*	
E. coli K-12 MG1655	*Proteobacteria*	Facultative	Negative	Stool (Palo Alto, CA)	Commensal	75.7	*acrD*, *acrE*, *acrF*, *bcr*, *emrA*, *emrB*, *emrD*, *emrE*, *emrK*, *emrY*, *mdfA*, *mdlA*, *mdlB*, *mdtA*, *mdtB*, *mdtC*, *mdtD*, *mdtE*, *mdtF*, *mdtG*, *mdtK*, *mdtL*, *mdtM*, *sdsP*, *sdsQ*, *sdsR*, *yojI*	*moaA*, *moaB*, *moaC*, *moaD*, *moaE*, *modA*, *modB*, *modC*, *moeA*, *moeB*, *mog*
K. pneumoniae	*Proteobacteria*	Facultative	Negative	Bloodstream (Washington University, St. Louis)	Opportunistic pathogen	9.8	*acrA*, *acrB*, *acrE*, *acrF*, *acrZ*, *emrA*, *emrB*, *emrD*, *mexA*, *mexB*, *mdfA*, *mdlB*, *mdtA*, *mdtB*, *mdtC*, *mdtD*, *mdtH*, *mdtK*, *mdtL*, *mdtM*, *mdtN*, *mdtO*, *oqxB19*, *stp*, *ybhF*, *ybhR*, *ybhS*, *yheI*	*moaA*, *moaB*, *moaC*, *moaD*, *moaE*, *moaF*, *modA*, *modB*, *moeA*, *moeB*, *mog*

a Frequency (%) computed from the gut-associated metagenome samples in the Human Microbiome Project II (48) (*n* = 553).

b Genes associated with molybdenum cofactor biosynthesis (22), including those described as critical for anaerobic deglycosylation of doxorubicin (13).

c Species confirmed from processing16S rRNA gene amplicon sequences in BLAST. Multidrug export and Moco biosynthesis genes were screened from the human microbiome project reference genome for E. faecium (GenBank Assembly accession no. GCA_000174395.2).

d Genes reported were based on whole-genome sequence assemblies for the isolates or, where indicated, reference genome.

**FIG 1:**
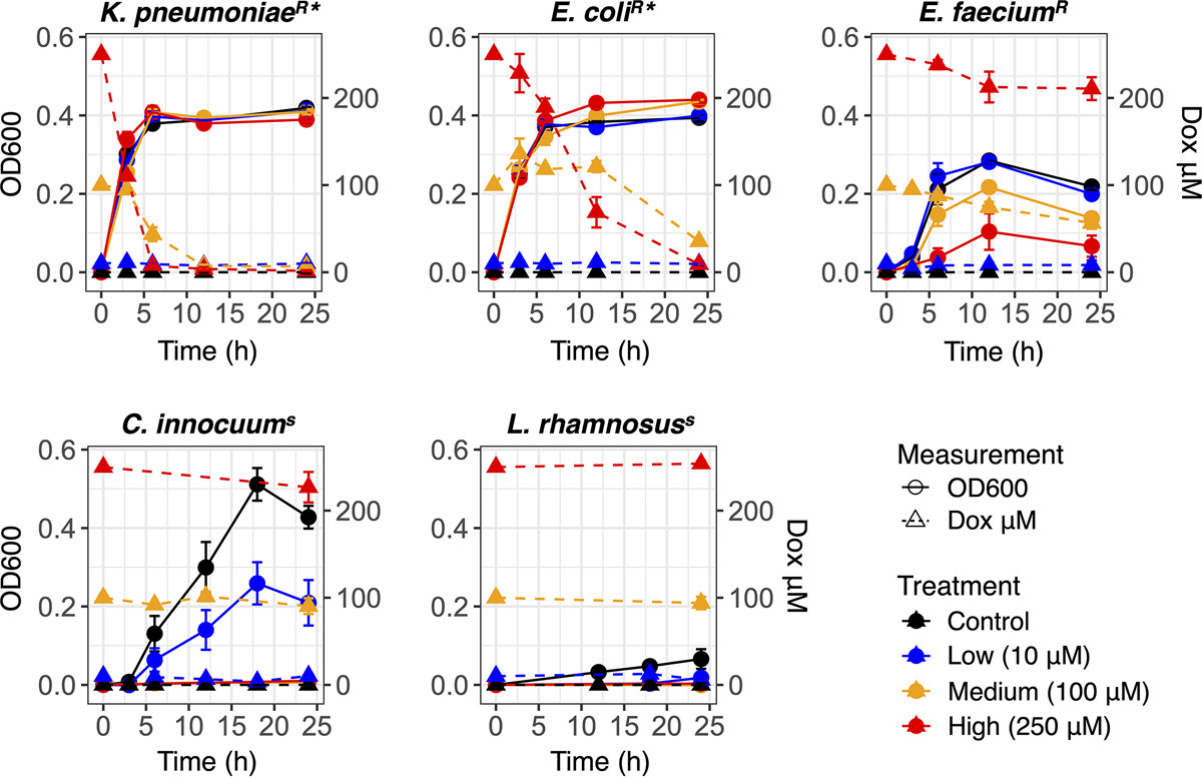
Bacterial resistance to and transformation of doxorubicin. Bacterial growth (circles and solid lines) and detected changes in doxorubicin concentration (triangles and dotted lines) in GAM media under anaerobic conditions. Superscript S, drug sensitive; R, drug resistant; *, drug transforming. Color corresponds to treatment of doxorubicin start concentration.

The predictions for bacterial drug resistance were validated with *in vitro* bacterial growth (optical density at 600 nm [OD_600_]) and drug transformation assays (Fig. 1). Bacteria were grown under anaerobic conditions in Gifu anaerobic broth media (GAM) supplemented with doxorubicin (0 μM [control], 10 μM, 100 μM, and 250 μM). K. pneumoniae and E. coli, the strains encoding putative doxorubicin transformation genes, were resistant to the drug at all concentrations. Consistent with the genomic analysis, both strains transformed the bioactive drug when exposed to 100 μM and 250 μM. Both strains did not appear to alter doxorubicin concentration when exposed to only 10 μM; the lack of detected change may be attributable to assay variation below 10 μM (Fig. S1 in the supplemental material). Nevertheless, under the higher drug exposures, concentration is positively associated with the onset time and rate of drug transformation. K. pneumoniae significantly reduced 250 μM and 100 μM within 3 and 6 h, while E. coli completed the similar process within 6 and 24 h; i.e., a linear mixed-effects model (LME) with fixed effects for treatment by time and a random effect for treatment indicated that these were the initial time points where detected drug concentration had become significantly different from that at time zero (*P < *0.05). The area under the curve (AUC) further demonstrated that K. pneumoniae was a more efficient drug transformer than E. coli (100 μM *P = *0.001; 250 μM *P = *0.004).

10.1128/mSphere.00068-21.1 FIG S1 (A) Standard curve linear regression for assay to detect doxorubicin (dox) concentration in GAM growth media. (B) Detection variation at low concentrations illustrated as the distribution for estimates of control samples (i.e., 0 μM dox, which the doxorubicin-containing sample estimates for concentration were normalized against) from the bacterial growth curves and mixed community experiment (*n* = 159; mean, −1.092; standard error, 0.376). Download FIG S1, TIF file, 0.6 MB. Copyright © 2021 Blaustein et al. 2021 Blaustein et al. https://creativecommons.org/licenses/by/4.0/ This content is distributed under the terms of the Creative Commons Attribution 4.0 International license.

E. faecium was also resistant to doxorubicin despite lacking the putative drug transformation genes. The highest drug concentration limited (*P < *0.05 for 250 μM at 6, 12, and 24 h) but did not abrogate its growth (Fig. 1), suggesting some level of drug tolerance via another resistance mechanism. While E. faecium did not appear to drastically lower drug concentrations like K. pneumoniae or E. coli, there was a detectable reduction of 100 μM doxorubicin by 24 h (*P = *0.005) and a small but nonsignificant reduction of 250 μM doxorubicin by 24 h (*P = *0.121) (Table 1). We note that significant abiotic degradation does not occur; the drug remains stable during incubation in GAM (Fig. S2).

10.1128/mSphere.00068-21.2 FIG S2 Stability of doxorubicin in GAM broth media. Sterile media containing doxorubicin was incubated with shaking at 37°C for 24 h. Download FIG S2, TIF file, 0.2 MB. Copyright © 2021 Blaustein et al. 2021 Blaustein et al. https://creativecommons.org/licenses/by/4.0/ This content is distributed under the terms of the Creative Commons Attribution 4.0 International license.

The slower-growing C. innocuum and L. rhamnosus, which also lacked putative doxorubicin-transforming genes and contained different sets of efflux genes than the other strains, were both drug sensitive (Fig. 1). Although C. innocuum had limited growth during the 10 μM drug exposure, its optical density remained significantly lower than that in the control media at 18 h (*P = *0.030) and 24 h (*P = *0.029).

In line with previous work (13), the doxorubicin transformation mechanism was dependent on anaerobic conditions. We found limited transformation of the drug by E. coli or K. pneumoniae when prepared and incubated for aerobic growth (Fig. S3). Although doxorubicin concentrations were altered by drug-resistant strains that were incubated aerobically in tryptic soy agar (TSA) by 24 h relative to controls (analysis of variance [ANOVA] *P = *0.001 for 100 μM; ANOVA *P = *0.031 for 250 μM), such changes were minimal compared to net reduction in drug concentration by the same strains in GAM under anaerobic conditions as illustrated in Fig. 1. Moreover, drug efflux provided protection against doxorubicin toxicity. The efflux pump inhibitor phenylalanine-arginine β-naphthylamide (PAβN) potentiated antimicrobial effects of the chemotherapeutic for all drug-resistant strains (ANOVA *P < *0.001; Fig. S4). Thus, efflux was the primary resistance mechanism for E. faecium, and the anaerobic process for doxorubicin biotransformation (i.e., E. coli and K. pneumoniae) appeared dependent on initial ability of the strain to resist chemical stress by effluxing the drug as well.

10.1128/mSphere.00068-21.3 FIG S3 Limited doxorubicin detoxification under aerobic conditions. Doxorubicin concentration in TSB after 12 h and 24 h of bacterial growth (or negative control) via incubation shaking at 37°C (tubes with loose caps for aeration). Values are normalized to controls containing no doxorubicin. Low (A), medium (B), and high (C) doxorubicin treatments are shown. Different letters above bars for a respective cluster indicate ANOVA *post hoc* Tukey’s *P* value of <0.05. Download FIG S3, TIF file, 1.4 MB. Copyright © 2021 Blaustein et al. 2021 Blaustein et al. https://creativecommons.org/licenses/by/4.0/ This content is distributed under the terms of the Creative Commons Attribution 4.0 International license.

10.1128/mSphere.00068-21.4 FIG S4 Efflux pump inhibitor phenylalanine-arginine β-naphthylamide (PAβN) potentiates antimicrobial effects of doxorubicin (dox). Drug-resistant strains were grown in GAM growth media supplemented with dox (*, 250 μM for K. pneumoniae and E. coli, 100 μM for E. faecium), PAβN, and both dox and PAβN. Colors correspond to treatment. Different letters above bars for a respective taxon indicate ANOVA *post hoc* Tukey’s *P* value of <0.05. Download FIG S4, TIF file, 1 MB. Copyright © 2021 Blaustein et al. 2021 Blaustein et al. https://creativecommons.org/licenses/by/4.0/ This content is distributed under the terms of the Creative Commons Attribution 4.0 International license.

### Bacteria-mediated detoxification restored growth of drug-sensitive strains.

**FIG 2:**
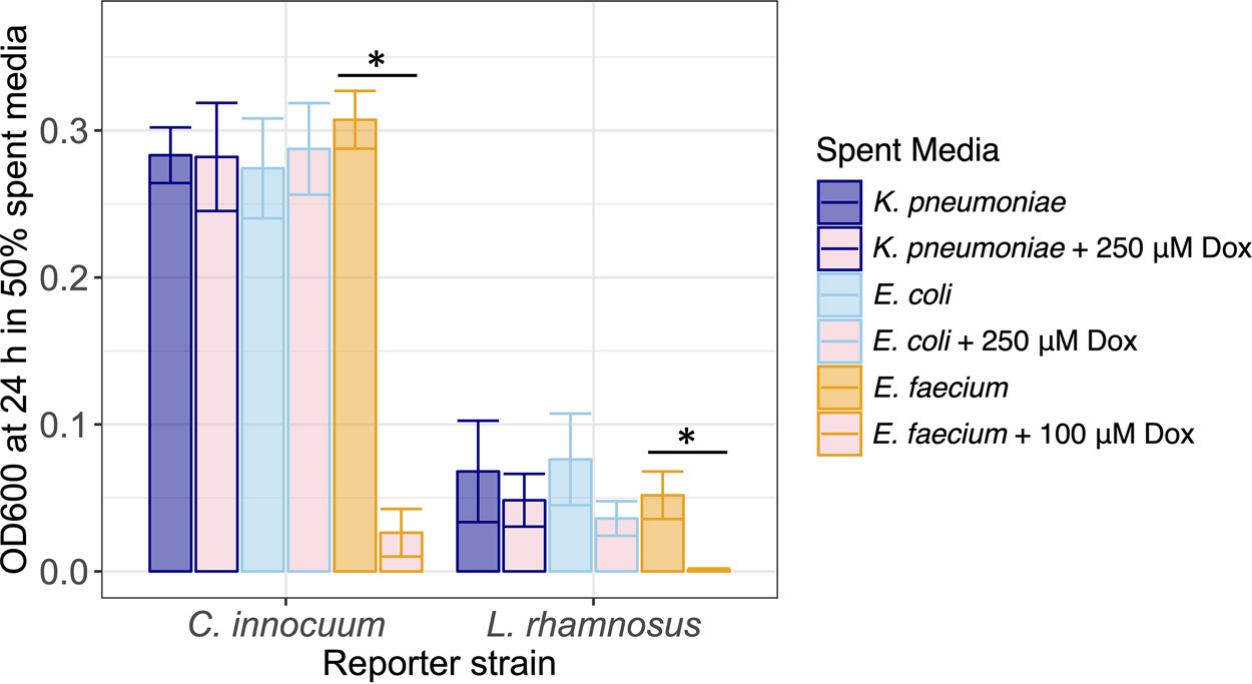
Growth of drug-sensitive bacteria (C. innocuum, L. rhamnosus) in spent media of drug-resistant strains (K. pneumoniae, E. coli, and E. faecium). The latter group had been grown with or without (control) doxorubicin. Color corresponds to spent media source. *, Mann-Whitney *P < *0.05.

To test if E. coli and K. pneumoniae would sufficiently detoxify doxorubicin to allow growth of drug-sensitive community members, each was grown under anaerobic conditions with and without drug. Filtration was employed to make cell-free spent media. Doxorubicin-sensitive C. innocuum and L. rhamnosus were subcultured in the cell-free spent media. Respective growth in filter-sterilized spent media of the drug-transforming strains (i.e., K. pneumoniae and E. coli) that were previously grown with and without doxorubicin was not significantly different (*P > *0.05 for all respective comparisons) (Fig. 2). In contrast, doxorubicin remained at a concentration in the cell-free spent media of E. faecium grown with the drug that remained inhibitory to growth of C. innocuum (*P = *0.002) and L. rhamnosus (*P = *0.035) relative to the respective controls (Fig. 2).

### Simplified bacterial community interactions with doxorubicin: overview.

**FIG 3:**
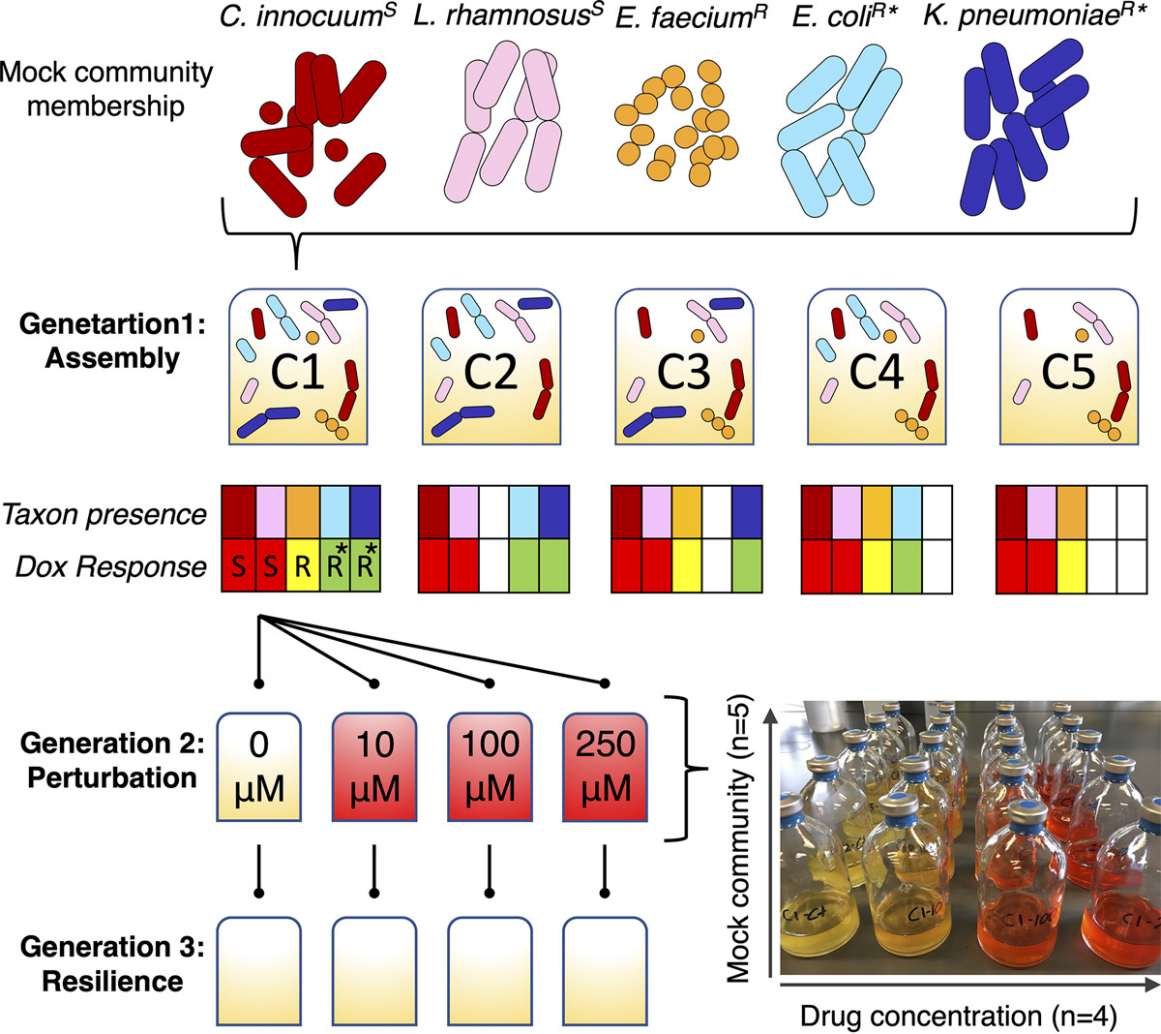
Experimental design for testing the effects of microbial community membership and doxorubicin concentration on drug transformation and resilience of the microbiota. Five taxa were selected for study as model members of the gut microbiota, each with a unique response to doxorubicin (see Fig. 1; superscript S, drug sensitive; R, drug resistant; *, drug transforming). Strains were pregrown and inoculated to form five different microbial communities using a reductionist approach (C1 to C5; community profile heatmaps indicate taxon presence/absence and response to doxorubicin). Each community was grown over three 24-h generations to evaluate assembly, perturbation, and resilience (e.g., the flowchart shows one replicate of C1 across three generations; the image shows one replicate of C1 to C5 in generation 2). The full experiment was performed in triplicate.

**FIG 4:**
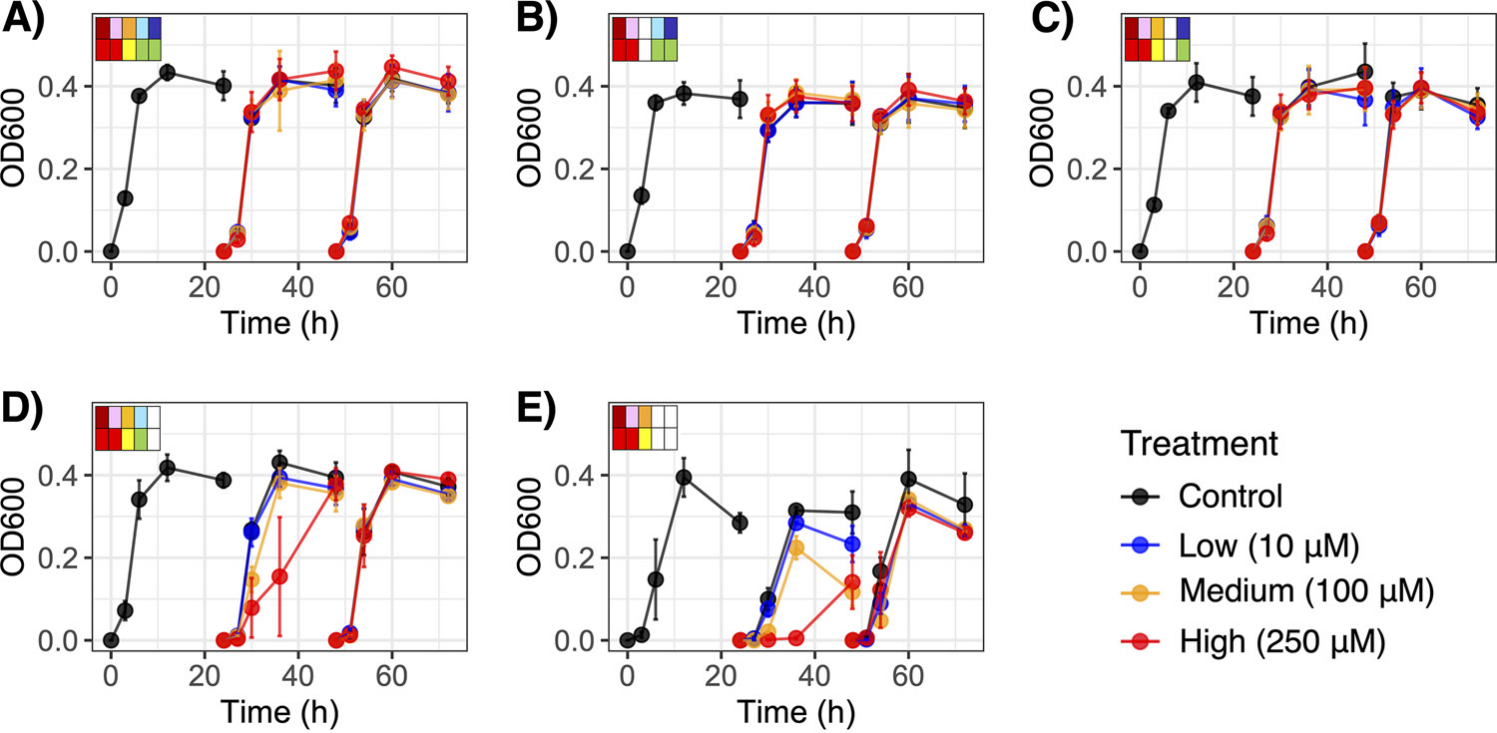
Changes in cumulative bacterial community growth (OD_600_) over time across three generations for the five microbial communities, C1 to C5 (A to E). The community profiles in the top corner of each panel indicate taxon presence/absence (top row) and response to doxorubicin (bottom row) as described in Fig. 3.

**FIG 5:**
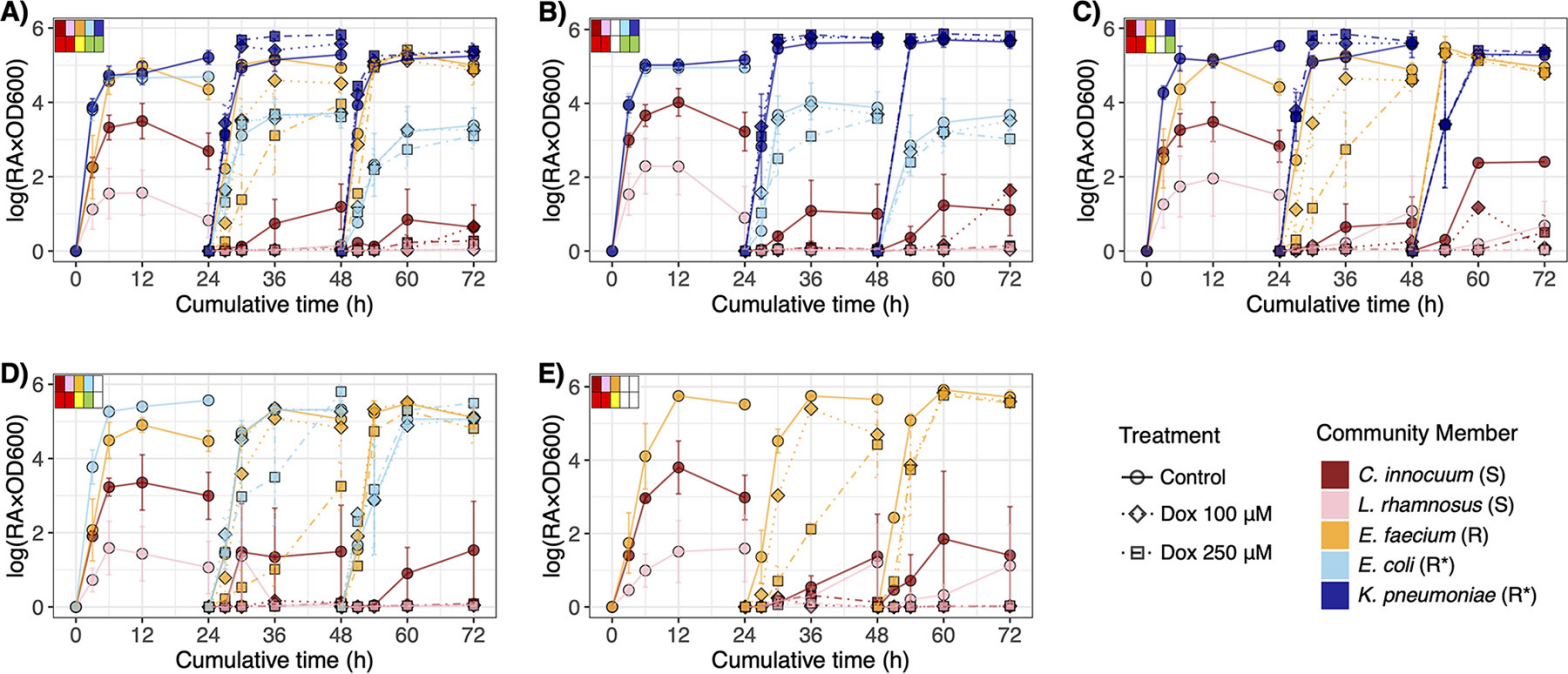
Relative growth of individual bacterial taxa (i.e., relative abundance of the taxon × OD_600_ as proxy for overall community density) within each microbial community, CI to C5 (A to E), over three generations (i.e., evaluating assembly, perturbation, resilience). Community profiles in the top corner of each panel indicate taxon presence/absence (top row) and response to doxorubicin (bottom row) as described in Fig. 3. Shape and line correspond to doxorubicin perturbation concentration. Data from the low treatment (Dox 10 μM) are presented in Fig. S5 in the supplemental material.

Model consortia were constructed to test the hypothesis that community membership (and associated functions) and chemotherapeutic exposure concentration impact drug detoxification and associated community resilience (Fig. 3). The OD_600_ was measured as proxy for cumulative bacterial growth (Fig. 4); 16S rRNA gene amplicon sequencing was used to determine changes in relative abundances of the specific strains within the mock bacterial communities over time. A total of 611 samples (*n* = 595 cocultures; *n* = 16 controls, including media control and positive and negative amplicon controls) were processed, from which there were 3,459,042 reads with classified taxonomic assignments. Of the classified reads, 99.6% matched the five target bacterial taxa, indicating that the starting consortia were negligibly contaminated, if at all. Each amplicon library contained 5,735 ± 167 reads (mean ± standard error). Counts per target taxon (i.e., the five bacterial strains) were converted to relative abundances and combined with the OD_600_ data to estimate relative growth of each individual strain over the three generations with respective doxorubicin treatments (Fig. 5; Fig. S5).

10.1128/mSphere.00068-21.5 FIG S5 Relative growth of individual bacterial taxa (i.e., relative abundance of the taxon × OD_600_ as proxy for overall community density) within each microbial community, CI to C5 (A to E), over three generations (i.e., evaluating assembly, perturbation, and resilience). Community profiles in the top corner of each panel indicate taxon presence/absence (top row) and response to doxorubicin (bottom row) as described in Fig. 3. Shape and line correspond to doxorubicin perturbation concentration. Download FIG S5, TIF file, 1.9 MB. Copyright © 2021 Blaustein et al. 2021 Blaustein et al. https://creativecommons.org/licenses/by/4.0/ This content is distributed under the terms of the Creative Commons Attribution 4.0 International license.

### Bacterial community baseline assembly and succession were stable.

Under baseline conditions with no doxorubicin treatment, community growth measured by OD_600_ varied based on bacterial community membership and largely reflected the lag time of the fastest-growing strain that was present (i.e., K. pneumoniae, E. coli, or E. faecium) (Fig. 4). According to an LME model with fixed effects for time by community and a random effect for community within generation 1 (G1), the bacterial communities C1 to C4 entered an exponential growth within 3 h and followed similar growth patterns (*P > *0.05 at all time points), while the OD_600_ of C5 remained lower at that time (*P = *0.022). Similar trends were observed in each subsequent generation (G2 and G3), though C5 still approached or reached stationary growth like the other communities by 12 h. Thus, all communities reached a well-defined stationary growth stage within the 24-h generation time (Fig. 4).

While the bacterial communities had been inoculated at relatively even strain concentrations in the range of 10^5^ to 10^6^ cells·ml^−1^ (ANOVA *P = *0.422) yielding similar initial 16S rRNA gene proportions (Fig. S6), the relatively fast-growing facultative strains consistently outcompeted C. innocuum and L. rhamnosus to establish a stable community structure where the latter two persisted at low relative abundances (Fig. 5). K. pneumoniae was generally the most successful strain under baseline conditions, followed by E. faecium and/or E. coli. The proliferation of K. pneumoniae even associated with minor suppression of E. coli (i.e., in C1 and C2), though not that of E. faecium (i.e., in C1 and C3) under control conditions (Fig. 5).

10.1128/mSphere.00068-21.6 FIG S6 (A) Estimated starting concentration of each strain in the respective mixed-microbial communities. (B) Average starting relative abundance detected in all communities. Download FIG S6, TIF file, 0.9 MB. Copyright © 2021 Blaustein et al. 2021 Blaustein et al. https://creativecommons.org/licenses/by/4.0/ This content is distributed under the terms of the Creative Commons Attribution 4.0 International license.

### Bacterial community membership affected transformation of doxorubicin (generation 2).

**FIG 6:**
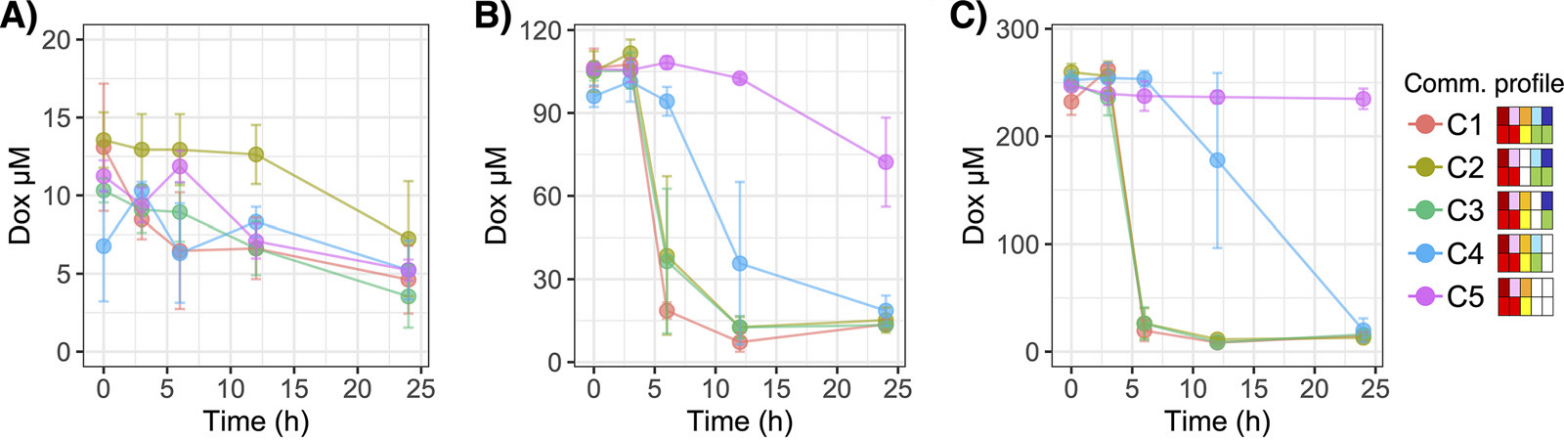
Changes in doxorubicin concentration during perturbation (i.e., generation 2 of continuous batch culture) for the five bacterial communities (C1 to C5). Ten micromolar (A), 100 μM (B), and 250 μM (C) doxorubicin treatments are shown. All values were normalized to the respective replicate control (community growth media with no doxorubicin). Color corresponds to community. The community profiles in the legend indicate taxon presence/absence (top row) and response to doxorubicin (bottom row) as described in Fig. 3.

The simplified communities were perturbed by their respective doxorubicin treatments in generation 2 (G2). While the C1 to C4 cocultures each contained at least one drug transformer strain (i.e., K. pneumoniae, E. coli, or both), drug resistance in C5 was putatively only conferred by efflux (i.e., E. faecium). As illustrated in Fig. 6, the AUC for doxorubicin concentration over time in G2 differed between C5 and C1 to C4 for the 100 μM treatment and between C5 and C1 to C3 for the 250 μM treatment (Tukey’s *P < *0.05 for each pairwise comparison). Trends for minor differences in AUC between C5 and C4 for the 250 μM treatment were notable as well (Tukey’s *P = *0.061). Likewise, LME models with fixed effects for treatment by time nested by repeated measures and a random effect for treatment for each community indicated that C1 to C3 and C1 to C4 significantly transformed 100 μM and 250 μM doxorubicin, respectively (*P < *0.001 for each respective treatment at 24 h). For C5, doxorubicin concentration did not change from 250 μM (*P = *0.319), though it was slightly but significantly lower at 24 h of G2 (i.e., 48 h overall) for the 100 μM treatment (*P =* 0.009) (Fig. 6). The small but significant reduction of 100 μM doxorubicin by C5 was similar to the observation for E. faecium in monoculture (see Fig. 1). Moreover, there were no detected changes in doxorubicin concentration for the 10 μM treatment for any bacterial communities (*P > *0.05 for all time points), which may reflect assay sensitivity for measurements below 10 μM (Fig. S1).

In line with expectations based on transformation previously observed in monoculture (Fig. 1), the K. pneumoniae-containing communities (C1 to C3) detoxified doxorubicin more rapidly than C4 in which E. coli was the only drug-transformer (Fig. 6). The AUC for the 250 μM treatment was significantly lower in C1 to C3 than C4 (Tukey’s *P < *0.05 for each pairwise comparison). In addition, comparing community-dependent drug transformation through application of LME models for fixed effects of community by time nested by repeated measures and a random effect for community indicated that drug transformation never significantly differed across C1 to C3 (*P > *0.05 at all time points for each respective treatment), while that by C4 was relatively limited for the 100 μM treatment through 6 h of G2 (*P < *0.001) and for 250 μM treatment through 12 h of G2 (*P < *0.001) (i.e., *P > *0.05 for each respective following time point). Moreover, the concentration of doxorubicin remained significantly greater in the C5 culture, which lacked drug-transforming strains, than all other communities throughout G2 (i.e., at 24 h, 100 μM *P = *0.005 and 250 μM *P < *0.001) (Fig. 6). Consistent with observations of monocultures of K. pneumoniae and E. coli (Fig. 1), the rate of drug detoxification appeared to positively associate with starting drug concentration. *Post hoc* pairwise comparisons in the LME model for estimated marginal means (EMM) indicated that C1 to C3 significantly reduced 250 μM doxorubicin relative to the nondrug-transforming C5 within 6 h of G2 (Tukey-adjusted *P < *0.001 for all pairwise comparisons). Alternatively, for the 100 μM treatment, only C1 demonstrated significant reduction compared to C5 within 6 h (Tukey-adjusted *P = *0.018), while the same was not reached until 12 h of G2 for C2 (Tukey-adjusted *P = *0.017) and C3 (Tukey-adjusted *P = *0.017). Overall, presence of particular drug-transforming strains drove the effects of bacterial community membership on doxorubicin detoxification. Since C1 (i.e., contained all strains) appeared to be slightly more efficient at drug transformation than C2 (i.e., all except E. faecium) or C3 (i.e., all except E. coli), at least for the 100 μM treatment, increasing community diversity may have possible synergistic impacts in promoting the primarily K. pneumoniae-mediated drug transformation.

### Chemotherapeutic exposure perturbed bacterial community membership (generation 2).

Doxorubicin exposures had significant effects on bacterial community growth, particularly suppressing C4 and C5 (Fig. 4). According to LME models with fixed effects for treatment by time nested by repeated measures and a random effect for treatment for each community, there were no significant effects of doxorubicin on the OD_600_ of C1 or C2 (*P > *0.05 for treatment across all time points), and the only difference for C3 was in the 10 μM treatment at 24 h of G2 (i.e., 48 h overall) (*P = *0.041). The 250 μM treatment repressed growth of C4 through 6 h (*P = *0.003) and 12 h (*P < *0.001) of G2, though the community rebounded by 24 h (*P = *0.832). In contrast, the OD_600_ of C5 was significantly lower at 24 h of G2 when exposed to 100 μM (*P *≤ 0.003) and 250 μM (*P *≤ 0.003) of doxorubicin. Such effects were attributable to the perturbation suppressing growth in C4 (i.e., where the only transformer was E. coli) and C5 (i.e., where there were no transformer strains). Thus, chemotherapeutic-induced growth suppression was dependent on both presence and efficacy of drug-detoxifying bacteria.

**FIG 7:**
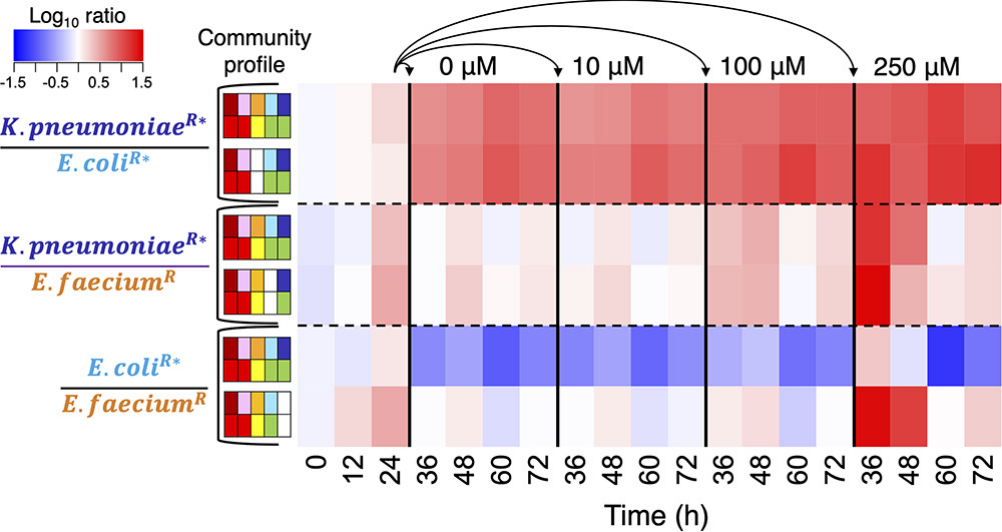
Ratios of the three drug-resistant bacteria (log-transformed average relative abundance proportions) in their respective microbial communities over time. Superscript S, drug sensitive; R, drug resistant; *, drug transforming. The community profiles indicate taxon presence/absence (top row) and response to doxorubicin (bottom row) as described in Fig. 3. At 24 h, microbial communities were serially transferred to new growth media containing the indicated concentration of doxorubicin (0 μM [control] to 250 μM). At 48 h, those microbial communities were further transferred to new growth media containing no drug.

Combining the OD_600_ with the 16S rRNA gene relative abundances as proxy for relative growth demonstrated that perturbation selected for the drug-resistant bacteria, especially the drug transformers. Both K. pneumoniae and E. coli experienced greater fitness during drug exposures (i.e., in G2) than E. faecium, where drug resistance was conferred by efflux. According to LME models, 250 μM doxorubicin had positive impacts on the relative growth of K. pneumoniae in C1 and C3 (*P < *0.05 at 6 to 24 h of G2 for both communities; Fig. 5) and its proportional abundances to E. faecium (*P < *0.05 for both communities; Fig. 7). In C1 and C4, the 250 μM doxorubicin treatment also had positive effects on proportional abundances of E. coli to E. faecium (*P < *0.05 for both communities) despite the former being somewhat limited by K. pneumoniae competition in C1 and by the drug exposure in C4 (*P = *0.050 at 12 h of G2) (Fig. 5; Fig. 7). Repression of E. coli growth in C4 under the high drug concentration was inconsistent with its observed resistance in monoculture (Fig. 1), suggesting that competition may have adversely impacted its fitness during perturbation. Moreover, relative growth of E. faecium was adversely impacted by 100 μM and 250 μM doxorubicin through 6 h and by the higher concentration through 12 h in all communities where it was present (i.e., C1, C3, C4, and C5) (*P < *0.05 for the respective treatments by time point in each community; Fig. 6). Although drug-resistant E. faecium still reached exponential growth in all communities during G2, its relative growth remained limited through 24 h of G2 (i.e., 48 h overall) in cultures that lacked drug-transforming K. pneumoniae (i.e., C4 and C5 *P < *0.05) (Fig. 5). Thus, fitness of E. faecium was positively impacted by drug transformation conferred specifically by K. pneumoniae (i.e., in C1 and C3, but not C4, where E. coli was the only drug transformer, or C5, where there were no transformers).

The two drug-sensitive strains became less abundant in all communities beyond G1, a reflection of having slower growth rates than counterpart strains (see Fig. 1) as well as lacking resistance to doxorubicin (Fig. 5). Such low abundances, including those that fell below the 16S rRNA gene sequencing detection range, yielded wide variation in G2. Nevertheless, according to the LME model, perturbation suppressed relative growth of C. innocuum at 24 h of G2 or C1 (100 μM *P = *0.018 and 250 μM *P = *0.026) and C5 (10 μM *P = *0.028, 100 μM *P* *=* 0.020, and 250 μM *P = *0.032) (Fig. 6). It had additional adverse impacts on relative growth of L. rhamnosus at 24 h of G2 in C1 (10 μM *P = *0.019 and 100 μM *P = *0.037), C2 (10 μM *P = *0.047), C3 (10 μM *P = *0.034, 100 μM *P* *=* 0.018, and 250 μM *P = *0.017), and C5 (100 μM *P* *=* 0.019 and 250 μM *P = *0.019). Therefore, despite somewhat limited data, growth suppression of all drug-sensitive strains by perturbation occurred in at least C1 and C5.

### Drug transformers conferred a protective effect on the broader community (generation 3).

In generation 3 (G3), residual doxorubicin was no longer present in the bacterial cultures since inoculum from each G2 had been transferred to fresh GAM media. The optical density of the previously growth-repressed communities (i.e., C4 and C5) no longer significantly differed based on treatment group in G3 (*P > *0.05 at all time points in the respective communities). Thus, removal of chemotherapeutic drug enabled the bacterial communities, at least the surviving strains, to rebound. For example, much like fitness of E. faecium benefitted from drug detoxification in G2, it was further restored upon residual drug removal via transfer to new media (*P > *0.05 for all respective treatments by time point beyond 6 h in each community in G3) (Fig. 5).

The low abundances and variation among drug-sensitive strains C. innocuum and L. rhamnosus, which reflected growth rate and doxorubicin sensitivity, were further exacerbated in G3. In fact, C. innocuum was only detected by quantitative PCR (qPCR) in 61.7% of the samples taken at the final time point (*n* = 37/60), perhaps reflecting dilution out of culture after the second serial transfer (i.e., G2 to G3). The C. innocuum data points for G3 from cultures where it had not been detected had to be voided as statistical replicates since regrowth potential could not be determined, limiting our analysis. In order to evaluate effects of treatment by community during a potential resilience stage (G3) for the drug-sensitive strains in C1 and C5, we compared controls (i.e., those that had received 0 μM doxorubicin in G2) to the respective communities that had received any doxorubicin exposure (10 to 250 μM). The aforementioned suppressive effect of doxorubicin on C. innocuum in G2 for C1 and C5 was only continued in G3 for C5 (i.e., the community lacking drug transformers; *P < *0.05 at all time points). Growth of C. innocuum was restored in C1 cultures (*P > *0.05 at all time points) in which both drug transformers had detoxified doxorubicin in the prior generation (Fig. 6). Perhaps an indication of resilience following drug transformation, additional trends were observed for C. innocuum regrowth in G3 for C1 to C3 that had been perturbed with at least 100 μM doxorubicin, though not for C4 and C5 (i.e., only communities containing the most efficient drug-transforming strain, K. pneumoniae) (Fig. 5). Thus, potential resilience of drug-sensitive strain populations to chemotherapeutic exposure appeared dependent on drug transformation by a keystone species.

## DISCUSSION

Antimicrobial use in medicine and agriculture has widely influenced the ecology of microbial communities that coexist in symbiotic association with human hosts. It is well established that antibiotics impair microbial diversity and enhance the dissemination of antimicrobial resistance genes (4, 16). Chemotherapeutics and nonantibiotic medicinal agents can also exert antimicrobial effects and cause alterations in the microbiome (5, 23). Conversely, human-associated microbial communities affect the fate of such drugs, from producing enzymes that mediate chemical transformations to modulating the metabolic activity of the host (24, 25). Thus, microbiome-drug interactions lead to dynamic changes across local biotic (e.g., modified community structure) and abiotic (e.g., limiting chemical stressors) microenvironments. Our *in vitro* simplified mixed communities exemplify some of these interactions through focusing on combinations of bacterial responses to doxorubicin such as cell death, drug efflux, and drug transformation.

Of these interactions, bacterial transformation of doxorubicin was most interesting, as it has the potential to affect not just the transforming organisms but also the surrounding community. The transformation could be metabolic or cometabolic, similar to bioremediation of antibiotics and other therapeutic drugs in the gastrointestinal tract (26, 27) and the environment (28, 29). The key distinction between these two strategies is determining whether the transforming organism itself directly benefits from the transformation by gaining energy or nutrients. In support of a metabolic mechanism, drug transformation provided competitive advantages to the two capable strains, especially the most efficient transformer (K. pneumoniae), relative to other members of the community. Additionally, the bacterial drug transformation response appeared more efficient as chemical stress increased (i.e., higher starting concentration). Since doxorubicin detoxification appeared to be strictly anaerobic, in line with a previous report (13), it may not be an energetically favorable process under aerobic conditions. The limited transformation we observed during aerobic incubation may have been due to anoxic microenvironments becoming established within suspended bacterial flocs. Future research focusing on microbiota-doxorubicin interactions under variable growth conditions (e.g., different substrates, minimal media, and oxygen transitions) is needed to better understand the mechanism for drug remediation applications.

While the drug-transforming strains received primary benefit from their doxorubicin response, biotransformation helped the broader microbial community as well. Detoxification *in vitro* supported resilience of drug-sensitive C. innocuum and improved fitness of drug-resistant but nontransforming E. faecium. The benefit to neighboring cells, a “bystander effect”(30), likely arose once the drug concentration in the homogenized media decreased to a tolerable concentration. As such, resilience of drug-sensitive strains appeared reliant on efficiency of transformation, as it did not occur when the process was relatively slow (i.e., in C4) or absent (i.e., in C5). Similarly, targeting biodegradation of chemical contaminants in the environment (e.g., herbicides) has been shown to restore native soil microbial community structure (31). Harnessing the beneficial potential of bacteria-mediated doxorubicin transformation to promote healthier intestinal microbiome diversity warrants further investigation. Compared to other antimicrobial resistance mechanisms in complex gut communities, this microbe-chemical interaction certainly appears more altruistic. Given the differential capabilities of K. pneumoniae and E. coli in drug transformation *in vitro*, despite putatively using the same mechanism, potential optimization for the promotion of microbiota resilience would require a better understanding of the full pathways involved as well as other factors, such as growth rate.

If we extrapolate our findings, we may expect to see similar bacteria-mediated doxorubicin metabolism in native intestinal microbial communities. Anaerobic deglycosylation of doxorubicin involves molybdenum cofactor biosynthesis (13), which is highly conserved among bacteria (22). However, since major side effects of cancer treatment with chemotherapy are gastrointestinal distress and mucositis (32, 33), the toxicity and antibiotic effects may outweigh protective effects of drug metabolism conferred by native microbiota. Much like in the natural environment, chemical transformations are common throughout the human gut, but perhaps not on a scale relevant for providing protective effects to patients undergoing treatment. Since the molybdenum cofactor is unstable (22), continuous metabolic activity for biosynthesis may be needed to confer anaerobic detoxification. Previous studies have reported that dietary prebiotics, such as inulin and oligofructose, may potentiate the effects of cancer therapies such as doxorubicin in cell culture and *in vivo* in mice with no added health risk (34, 35). An interesting avenue for future research would be to focus on improving strategies for biostimulation or bioaugmentation of doxorubicin detoxification conferred by the drug-transforming strains to limit dosage and gastrointestinal side effects.

Probiotic therapeutic interventions are extraordinarily popular and increasingly marketable; they are the third most commonly used natural product for both adults and children (36, 37). However, probiotic interactions within the human microbiome remain poorly understood, which leads to difficulty in implementing effective interventions. Since intravenously administered doxorubicin is known to cycle through the bloodstream to complete intended antitumor effects before residually accumulating in the gut (38), we hypothesize that the probiotic strain(s) may reduce overall drug toxicity (and mitigate antibiotic impacts on the microbiome) without limiting essential therapeutic bioavailability. While bacteria-mediated anaerobic doxorubicin detoxification has been shown to lower toxicity on the model organism C. elegans (13), the metabolites may still yield damaging impacts in human tissue (33). Thus, to extend this work with translational applications, the spectrum of microbiome-host-chemical interactions during doxorubicin treatment needs to be better understood. The conceptual model for microbiota resilience in this study was limited to an *in vitro* framework on microbial community interactions with the chemotherapeutic. It remains unclear whether biotransformation of doxorubicin may provide protective effects on microbiota *in vivo*, where there would be impacts from host cell interactions as well as different microniches (i.e., in contrast to homogenized growth media). Nevertheless, our findings have implications for developing probiotics to provide gut-specific protection to patients treated with doxorubicin and, more broadly, anthracycline chemotherapeutics.

## MATERIALS AND METHODS

Interactions between model human-associated bacteria and doxorubicin were investigated *in vitro*, with the primary focus to determine effects of drug concentration and bacterial community membership on drug transformation and resilience of drug-sensitive strains.

### Genomic and phenotypic assessments of doxorubicin resistance.

Our scope included a mix of facultative and strict anaerobes representative of normal human flora and opportunistic pathogens, each of which had a unique predicted response to doxorubicin (i.e., C. innocuum, E. coli, E. faecium, K. pneumoniae, and L. rhamnosus) (Table 1). Pure cultures of each strain were maintained in glycerol stocks (25%) and cultivated on Gifu anaerobic broth media (GAM; HiMedia) and tryptic soy agar (TSA). GAM was used since it supports recovery of overall community structure in gut microbiota most similar to observations with cultivation-independent approaches (39). Throughout the study, all strains were processed for growth under anaerobic conditions in a UNILab Pro anaerobic glove box (MBraun) circulated with zero-grade N_2_ unless otherwise indicated.

The DNeasy Powersoil kit (Qiagen) was used to extract isolates’ DNA, and whole-genome sequencing (Illumina HiSeq; 2 × 150 cycles) was performed at the Broad Institute Genome Services Center. Sequence data were preprocessed for quality control with TrimGalore v0.4.4 (https://www.bioinformatics.babraham.ac.uk/projects/trim_galore/) using a minimum phred score of 30, assembled *de novo* with SPAdes v3.12.0 (40), evaluated for quality control with CheckM v1.0.7 (41), and annotated with prokka v1.14.5 (42). Annotated genomes were screened for gene products associated with the resistance to doxorubicin and/or daunorubicin, multidrug efflux, and anaerobic deglycosylation of doxorubicin (i.e., *moaA*, *moaD*, *moeA*) (13) and the related molybdenum cofactor biosynthesis pathway (22).

Following genomic prediction, potential antibiotic effects of doxorubicin on each strain were characterized. The facultative bacteria were initially grown on TSA plates incubated at 37°C (E. coli and K. pneumoniae for 24 h, E. faecium for 48 h, and L. rhamnosus for 72 h). C. innocuum was grown on GAM plates sealed in AnaeroPack jars supplemented with gas generator sachets (Fisher Scientific) at 37°C for 24 h. The pure cultures were then set in the anaerobic chamber, and inoculum (i.e., three isolate colonies) was transferred via sterile wooden stick into sterile 15-ml glass Balch tubes containing 4 ml of GAM broth media supplemented with doxorubicin (control, 0 μM; low, 10 μM; medium, 100 μM; and high, 250 μM). Tubes were sealed with butyl rubber stoppers, crimped with aluminum caps, and placed in a 37°C shaking incubator. Culture samples were collected at 0, 3, 6, 12, and 24 h. Sample collection involved using a syringe BD PrecisionGlide needle (Sigma) needle to pierce the rubber caps of inverted tubes (after disinfecting the cap with 70% ethanol) and carefully drain approximately 250 μl of liquid media into a sterile Eppendorf tube. Additionally, growth responses of the facultative strains to the same doxorubicin exposures were evaluated under aerobic conditions grown in TBS media in 15-ml Falcon tubes with loosened caps and incubated with shaking at 37°C.

The Synergy HTX Multi-Mode microplate reader (Biotek) was used to measure the optical density of each sample at 600 nm (i.e., proxy for bacterial growth) and, following centrifugation at 12,000 × *g* for 1 min, 480 nm (i.e., proxy for doxorubicin concentration). The latter was our adapted spectrophotometric assay based on previous studies that indicated that doxorubicin transformation to less toxic metabolites in liquid media involves solution color change (13), and its concentration associates with absorbance at 480 nm (43) (Fig. S1 in the supplemental material). All absorbance measurements were normalized to controls of sterile GAM media.

We further evaluated whether efflux pump inhibitor phenylalanine-arginine β-naphthylamide (PAβN) potentiated antibiotic effects of doxorubicin on bacteria with observed resistance. Strains were grown anaerobically at 37°C for 24 h in GAM broth containing PaβN at 25 g·ml^−1^ supplemented with or without (control) doxorubicin. Taken together with the bacterial growth responses under aerobic/anaerobic conditions described above, potential dependencies of doxorubicin resistance and the drug transformation on drug efflux were determined to better understand the mechanism.

The genomic screen and phenotypic assays enabled classification of the select strains as doxorubicin sensitive, resistant (efflux), or transforming. Potential protective effects of doxorubicin transformation on drug-sensitive strains were evaluated in assays using spent media, i.e., drug-resistant strain growth media that still contained any residual doxorubicin and/or metabolites. The drug-resistant strains were grown anaerobically in 25 ml GAM broth in sterile 100-ml glass serum bottles supplemented with the highest drug concentration where resistance had been observed. Cultures were prepared in the anaerobic chamber and incubated with shaking at 37°C for 24 h as described above. The spent medium was processed by centrifugation and filter sterilization (0.2 μm filters) to remove bacterial cells. The cell-free medium was moved back into the anaerobic chamber to be reduced via atmospheric exposure for 24 h. Growth of drug-sensitive strains was assessed in 4 ml of 50:50 spent media/fresh media at 37°C for 24 h. Final optical density (600 nm) and doxorubicin concentration were measured as described above.

### Bacterial interactions in mixed communities.

Simplified bacterial communities were constructed to test the hypothesis that gut microbiota membership (and associated functions) and therapeutic concentration impact rates of drug detoxification and associated microbiota resilience. A 4 by 5 factorial design was applied in which doxorubicin concentration (0 μM, 10 μM, 100 μM, and 250 μM) and bacterial community composition (i.e., drug-sensitive strains mixed with five different combinations of drug-resistant and drug-transforming bacterial strains) varied (Fig. 3). All treatments were evaluated in triplicate.

Mixed bacterial communities were assembled and grown in continuous anaerobic batch cultures over three generations (referred to as G1, G2, and G3) to assess community assembly (G1), response to doxorubicin exposure (G2), and potential resilience following perturbation (G3) (Fig. 3). Strains were pregrown anaerobically in 25 ml GAM broth in sterile 100-ml glass serum bottles to reach log or early stationary phase and then transferred back to the anaerobic chamber to establish community assignments (i.e., all G1s for C1 to C5). Each mixed community was also grown anaerobically in 25 ml GAM broth in sterile 100-ml glass serum bottles and began with a combined inoculum of its respective strains in the range of 10^5^ to 10^6^ CFU·ml^−1^. Plate counts on GAM media incubated in AnaeroPack jars were used to confirm the starting concentration of each strain. G1 was incubated with shaking at 37°C for 24 h, and culture samples were collected at 0, 3, 6, 12, and 24 h with the syringe method described above. The G1 bottles were then transferred back to the anaerobic chamber, and a 20-μl aliquot was used to inoculate G2. The G2 bottles contained GAM broth supplemented with 0 μM (control), 10 μM, 100 μM, and 250 μM doxorubicin. These cultures were incubated and sampled the same way, and then a 20-μl aliquot was used to inoculate G3 (i.e., media without any drug supplement). All samples were processed to determine cumulative bacterial growth (optical density) and doxorubicin concentration (G2 samples only) as described earlier, and the remaining sample content was stored at −20°C.

### Molecular processing.

G1 to G3 samples were processed for 16S rRNA gene sequencing to characterize changes in bacterial community composition and structure over time as a factor of treatment. Samples were thawed, and DNA was extracted with MagAttract PowerMicrobiome DNA/RNA EP kits (Qiagen) using the epMotion 5073 automated platform (Eppendorf). 16S rRNA gene amplicons were generated using a slightly modified Earth Microbiome Project protocol (49). PCR was performed using 515f and 806r (barcoded) primers in duplicate 10-μl reaction mixtures with *Taq* DNA polymerase in 384-well plates. Positive controls were E. coli K-12 isolate DNA, and negative controls did not contain a DNA template. Plates were stored at −20°C until further processing.

Amplicon DNA concentration was estimated via densitometry. Duplicate PCR products were combined and run on 1.5% agarose gel containing ethidium bromide and imaged via G:Box Chemi XX6 (Syngene). ImageJ software (44) was used to estimate relative amplicon concentration by normalizing to band intensity of amplicon products to the 400-bp band of ladder DNA. Amplicons were pooled at putative equimolar concentrations and cleaned with the QIAquick PCR purification kit (Qiagen).

Sequencing was performed on Illumina MiniSeq (2 × 150) at the University of Illinois at Chicago Sequencing Core. Demultiplexed sequence data were analyzed using qiime2 v2018.8.0 (45). Paired-end sequences were preprocessed and denoised to generate amplicon sequence variants (ASVs) that were characterized with the silva-132-99-nb-classifier.

Due to dilution factors from G1 to G3 yielding potential ASV counts outside the detection range (i.e., cases where there was relatively minimal growth prior to transfer across generations), we also used qPCR to track the presence of C. innocuum in the final samples (i.e., G3; time, 24 h). Primers were designed in NCBI Primer-BLAST to target a 112-bp region of *yunB* (forward, GCCAAGCAGCATACAGGGTT; reverse, TATCCCCGTTCTTTGTGCGAT), i.e., a single-copy functional gene of C. innocuum (encoding sporulation protein YunB) that was not present in any other members of the bacterial community based on screening whole-genome sequence data. Minimal cross-amplification with nontarget strains from the mixed bacterial communities was validated using standard PCR. We performed qPCR in duplicate 25-μl reaction mixtures on the QuantStudio 3 system using PowerUp SYBR Green master mix (Applied Biosystems) with the protocol adjusted for a 58°C optimal melt temperature. Threshold cycle (*C_T_*) values were converted to CFU estimates based on the dilution series from DNA extraction.

### Statistical analysis.

Statistical analyses and data visualization were performed in R v3.6.0. A linear mixed-effects model was applied to characterize changes in bacterial growth (OD_600_) and doxorubicin transformation by individual strains, i.e., setting a fixed effect for time by treatment (doxorubicin exposure concentration) and a random effect for treatment. Differences in doxorubicin transformation efficiency across the drug-transforming strains were determined by comparing area under the curve with the Student's *t* test. For all drug-resistant strains, ANOVA with Tukey’s *post hoc* test was applied to determine changes in doxorubicin concentration under aerobic conditions and changes in bacterial growth under efflux pump inhibition. The Mann-Whitney test was further applied to evaluate restoration of drug-sensitive strain growth in spent media of drug-resistant strains that had either been grown with or without doxorubicin.

For the mixed microbial communities, ANOVA was used to evaluate potential differences in inoculum concentrations. Differences in doxorubicin transformation efficiency across communities were determined by comparing area under the curve with ANOVA and Tukey’s *post hoc* test. Linear mixed-effects models were applied to determine differences in overall bacterial growth (i.e., OD_600_), doxorubicin concentration, individual strain relative abundance proportions, and individual strain relative growth [i.e., log(OD_600_ × relative abundance)] over time, setting fixed effects for community and/or treatment by time nested by repeated measures and random effects for community and/or treatment. For *post hoc* pairwise comparisons, estimated marginal means for factor combinations were computed. Since log transformation of “zero” values is undefined, bacteria in G1 and G2 samples that had a 0% relative abundance (i.e., below detection range) were tallied as present with a relative abundance equivalent to 50% that of a singleton in the sample (46, 47). Due to the relatively low detection range for C. innocuum, especially by G3 (i.e., due to inherent dilution across the two generation transfers), only G3 samples with 0% relative abundance and detection via qPCR were treated this way. Alternatively, C. innocuum values in G3 samples with a 0% relative abundance and a negative qPCR result were excluded from statistical analysis since it could not be confirmed whether the taxon was present below the detection range or truly absent. For all statistical tests, α was set at 0.05.

### Data availability.

The genome assemblies of the isolates and the 16S rRNA gene amplicon sequencing data for the mixed community samples are available under NCBI BioProject accession no. PRJNA716959. All data and code that may be used to reproduce our analyses are available at https://github.com/hartmann-lab/bacteria_doxo_interactions.
