# Supplementary figures and images for: Biotransformation of Doxorubicin Promotes Resilience in Simplified Intestinal Microbial Communities
Source: mSphere. 2021 May 26;6(3):e00068-21. doi: 10.1128/mSphere.00068-21 (PMC8265622; doi:10.1128/mSphere.00068-21)

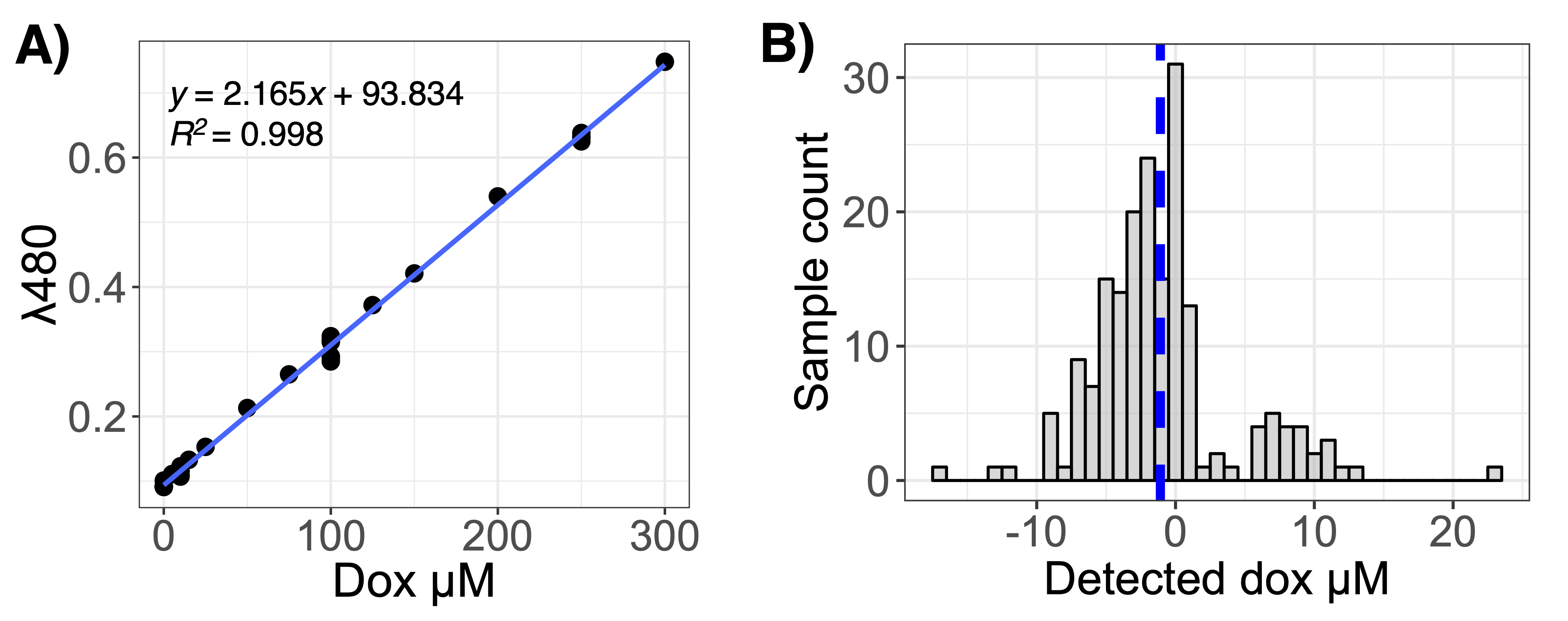

Supplement: FIG S1 [file msphere.00068-21-sf001.tif]

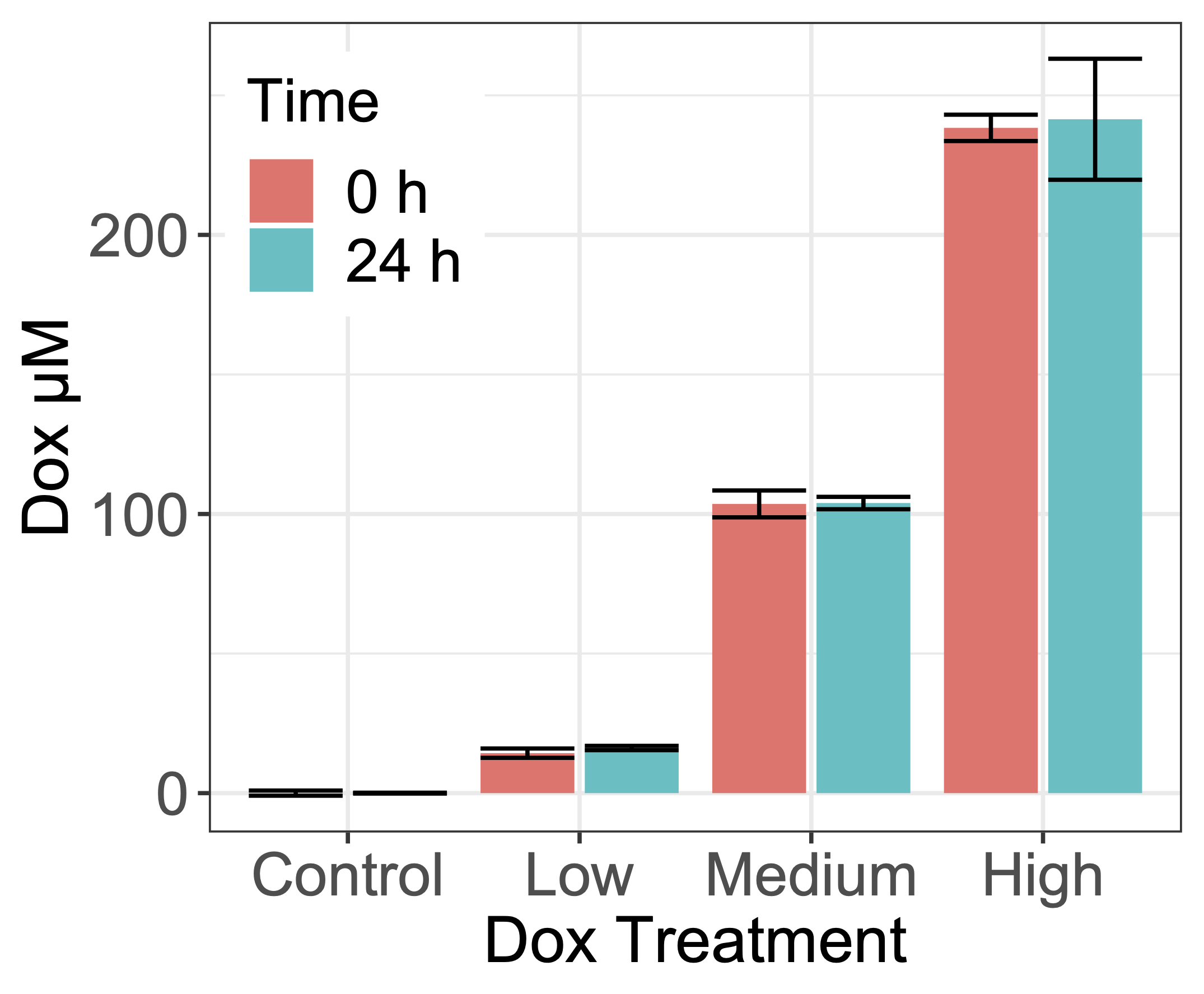

Supplement: FIG S2 [file msphere.00068-21-sf002.tif]

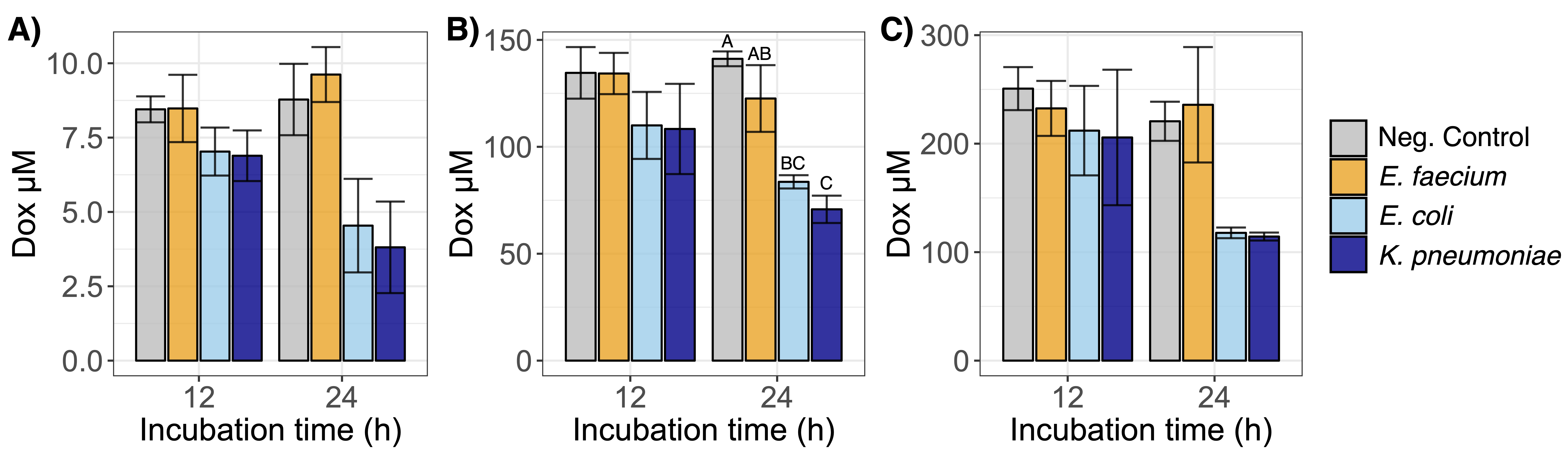

Supplement: FIG S3 [file msphere.00068-21-sf003.tif]

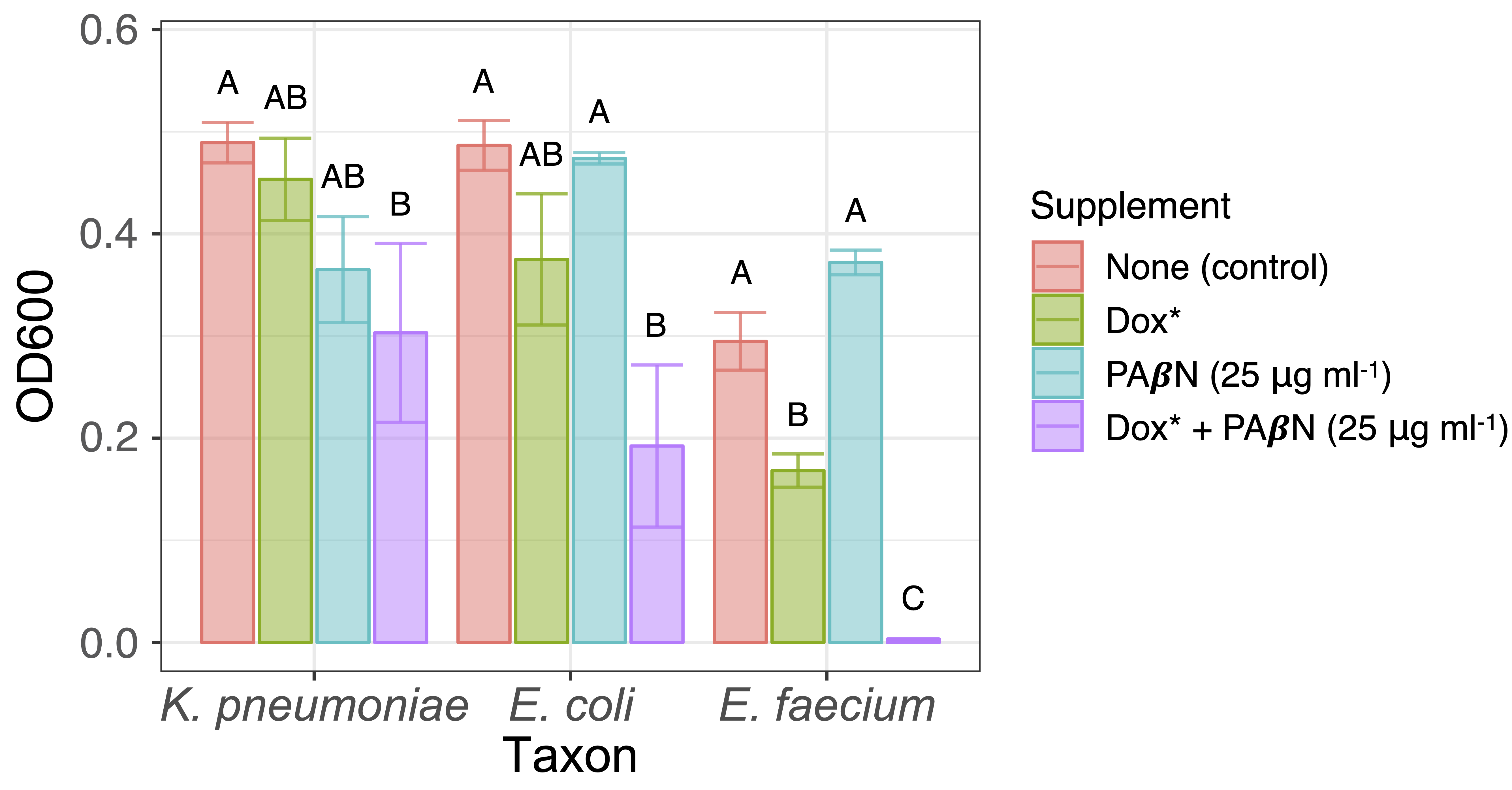

Supplement: FIG S4 [file msphere.00068-21-sf004.tif]

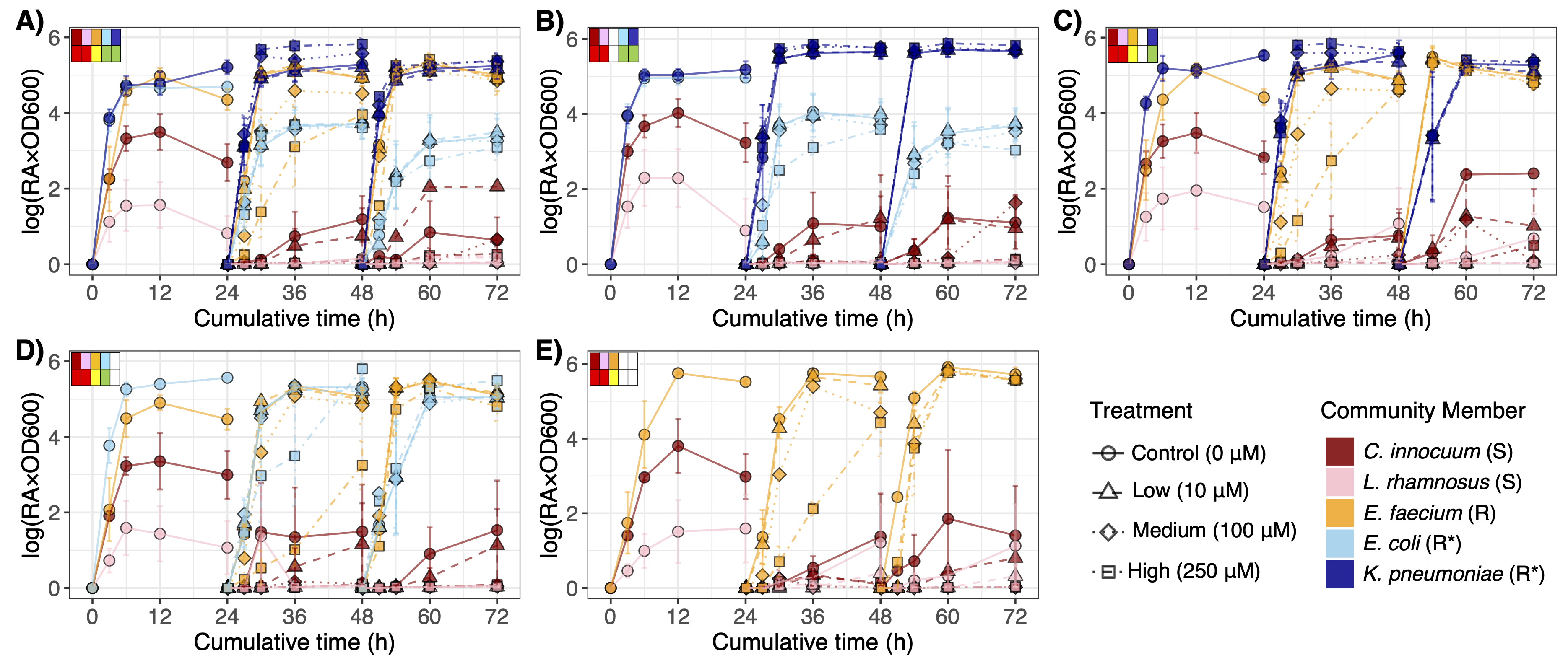

Supplement: FIG S5 [file msphere.00068-21-sf005.tif]

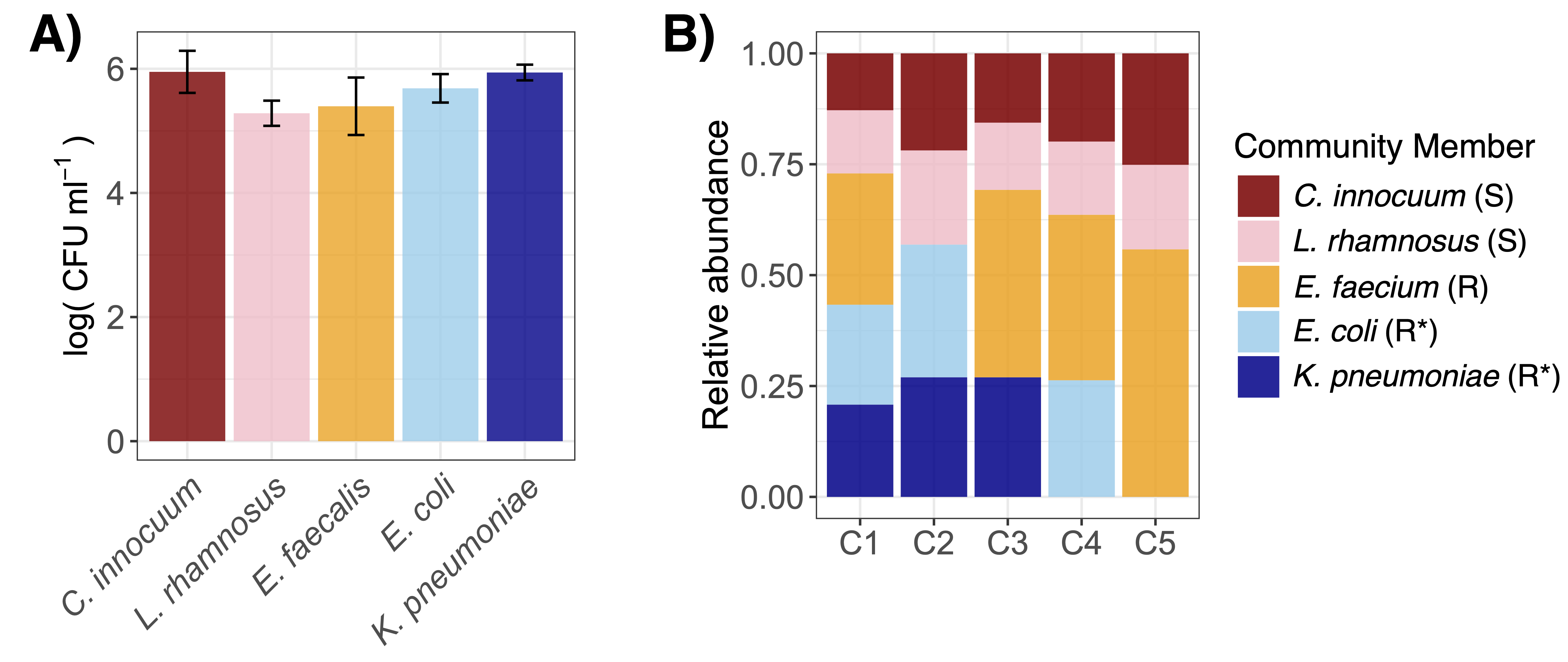

Supplement: FIG S6 [file msphere.00068-21-sf006.tif]
